# Isolated Blunt Pancreatic Head Injury with Evolving Acute Peripancreatic Fluid Collection in a Child Successfully Managed Conservatively

**DOI:** 10.3390/pediatric18020042

**Published:** 2026-03-17

**Authors:** Dumitru Marius Dănilă, Cristina-Mihaela Popescu, Irina Profir, Ada Ștefănescu, Gabriela Gurău

**Affiliations:** 1 School for Doctoral Studies in Biomedical Sciences, “Dunărea de Jos” University of Galați, 800008 Galati, Romania; marius_danilla@yahoo.com; 2 “Sf. Ioan” Clinical Emergency Pediatric Hospital, 800487 Galati, Romania; gabriela.gurau@yahoo.com; 3 Department of Dental Medicine, Faculty of Medicine and Pharmacy, “Dunărea de Jos” University of Galați, 800201 Galati, Romania; ada.stefanescu@ugal.ro; 4 Clinical Medical Department, Faculty of Medicine and Pharmacy, “Dunărea de Jos” University of Galați, 800216 Galati, Romania; 5 Department of Morphology and Functional Sciences, Faculty of Medicine and Pharmacy, “Dunarea de Jos” University of Galați, 800008 Galati, Romania

**Keywords:** blunt abdominal trauma, conservative treatment, pancreatic pseudocyst, pediatric pancreatic trauma, traumatic pancreatitis

## Abstract

**Background**: Pancreatic trauma (PT) in children is rare and associated with significant morbidity. The optimal form of management—operative versus non-operative—remains controversial, particularly in the presence of acute post-traumatic peripancreatic fluid collection, which may later evolve into pancreatic pseudocysts. Isolated pancreatic injuries without associated organ damage are uncommon and pose diagnostic and therapeutic challenges. **Case Presentation**: We report a 5-year-old boy who sustained an isolated grade IB blunt pancreatic head contusion following blunt abdominal trauma after falling onto a wooden fence. He presented with epigastric pain, repeated emesis, and an abdominal wall bruise. Initial ultrasound (US) findings were subtle; however, serial imaging and contrast-enhanced computed tomography (CECT) revealed focal contusion of the pancreatic head/uncinate process with a small peripancreatic fluid collection. Pancreatic enzymes were markedly elevated, with peak serum lipase reaching approximately 6579 U/L. The child remained hemodynamically stable and was managed conservatively with bowel rest, intravenous fluids, octreotide, proton-pump inhibition, pancreatic enzyme replacement therapy (PERT), and antibiotics. Serial US demonstrated the dynamic evolution of an acute peripancreatic fluid collection (APFC) (~2 cm), which remained stable without complications. Clinical and biochemical parameters gradually improved, and no invasive intervention was required. The patient was discharged on hospital day 16 with planned outpatient imaging follow-up. **Conclusions**: This case demonstrates that isolated pediatric pancreatic contusions complicated by small, evolving peripancreatic fluid collections can be safely managed non-operatively in hemodynamically stable patients. Serial ultrasound plays a key role in monitoring lesion evolution and guiding management decisions. In accordance with current pediatric trauma guidelines, careful observation with structured follow-up may prevent unnecessary invasive interventions while achieving excellent clinical outcomes.

## 1. Introduction

Blunt pancreatic injury (BPI) in children is an uncommon entity, occurring in approximately 3–12% of pediatric blunt abdominal trauma cases [1]. The most frequent mechanism is direct epigastric impact, classically from bicycle handlebars or similar objects, accounting for up to 50% of cases [1]. Pancreatic injuries in children are associated with significant morbidity, especially when diagnosis is delayed or when ductal injuries are missed [1,2]. The small size and retroperitoneal location of the pediatric pancreas make the detection of injury challenging [3].

Unlike adults, who often undergo operative intervention, there is no universal consensus on pediatric management; some centers favor non-operative management (NOM) even for major pancreatic injuries, while others advocate early surgery in high-grade injuries [4]. BPI can lead to complications such as acute pancreatitis or peripancreatic fluid collection. In accordance with the revised Atlanta Classification, early peripancreatic fluid collections are designated as APFCs, which may progress over four or more weeks into pancreatic pseudocysts (PPCs)—encapsulated fluid collections defined by a well-formed inflammatory wall [1,2,5].

We present a case of an isolated posttraumatic pancreatitis in a child, complicated by an evolving APFC, managed successfully without surgical intervention. Diagnosis was established, corroborated by imaging and laboratory tests [6]. This report aims to illustrate the diagnostic pathway and non-operative therapeutic strategy in pediatric pancreatic trauma, with a focus on the clinical significance of peripancreatic fluid collections and their evolution.

## 2. Materials and Methods

This case report was prepared in accordance with the CARE guidelines for clinical case reporting. Patient information was obtained from hospital records and imaging studies. Informed consent for publication of this case was obtained from the patient’s father. Relevant articles on pediatric pancreatic injury management, outcomes, and the evolution of APFC were reviewed and are cited to support the discussion. Approval from the Ethics Committee of “Sf. Ioan” Clinical Emergency Pediatric Hospital in Galați was obtained.

## 3. Case Presentation

### 3.1. Patient Information and Presentation

A 5-year-and-2-month-old male with no significant medical history was brought to the emergency department after a blunt abdominal trauma (BAT). The injury occurred when he fell and struck his upper abdomen on the edge of a wooden fence. The child immediately developed epigastric abdominal pain and repeated emesis. On examination, he was alert but in moderate distress, with ecchymosis and superficial abrasions across the upper abdomen. Vital signs were within age-appropriate limits (heart rate ~120/min, blood pressure 100/60 mmHg, afebrile), except for mild tachycardia attributable to pain. Abdominal examination revealed mild distension and epigastric tenderness without guarding or rebound. Bowel sounds were present. No other injuries were noted on secondary survey (no head trauma or orthopedic injuries). Initial tests are detailed in the next subsection. Key laboratory trends are summarized in Table 1.

### 3.2. Laboratory Results

**Figure 1:**
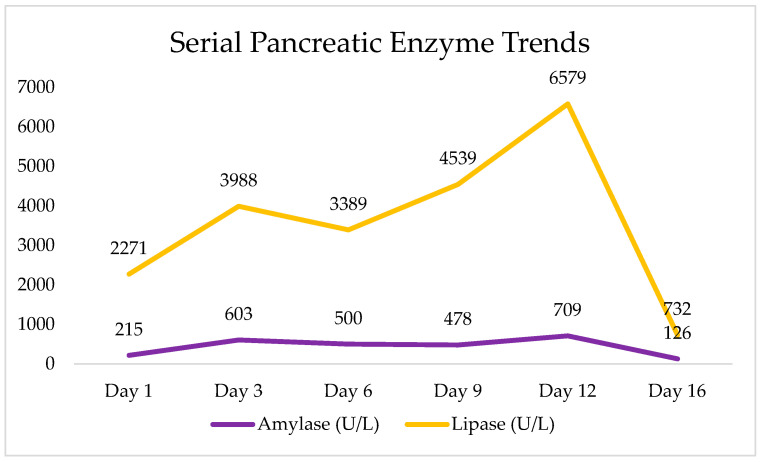
Serial serum pancreatic enzyme levels (amylase and lipase) throughout the hospitalization period.

**Figure 2:**
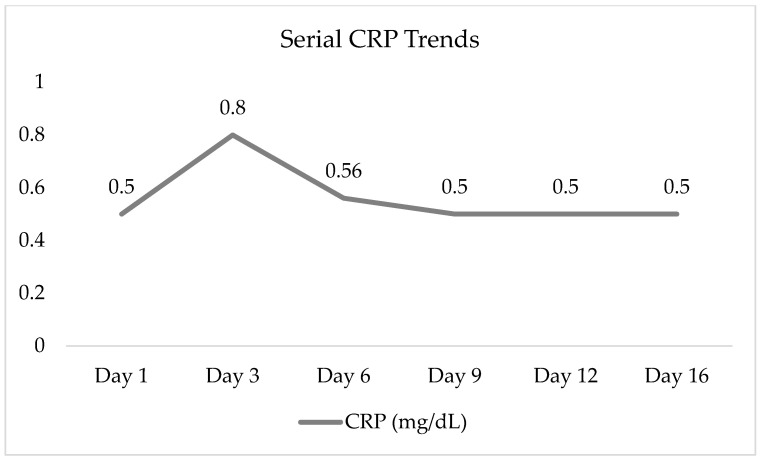
Serial C-reactive protein (CRP) levels throughout the hospitalization period. Values below 0.5 mg/dL are reported as 0.5, consistent with the assay’s lower detection limit.

Key laboratory trends are summarized in Figure 1 and Figure 2, and in Table 1.

Initial laboratory tests showed an elevated white blood cell count of 14 × 10^3^/μL (normal 4–12), mildly elevated liver enzymes (AST 67 U/L, ALT 48 U/L), and markedly elevated pancreatic enzymes, with serum amylase at 215 U/L (normal 30–110) and lipase at 2271 U/L (normal 23–300). These findings were consistent with post-traumatic pancreatitis. Hemoglobin was stable at 12.4 g/dL, and other parameters were within normal ranges. Serum amylase peaked at 709 U/L by day 12. Serum lipase rose to a peak of 6579 U/L on day 12. Both enzymes trended downward by the third week (amylase: 126 U/L; lipase: 732 U/L on day 16). Inflammatory markers were only modestly elevated: CRP was <0.5 mg/dL on day one, rose to 0.8 on day three, then normalized (<0.5) by day nine. The WBC, initially 14 × 10^3^/μL on day one, down-trended to 7.0 by day 6 and was within normal range thereafter (5.1 × 103/μL by day 16). Hemoglobin remained stable (~12.4–13.0 g/dL) throughout with stable hematocrit, indicating no significant internal bleeding. Mild transient elevations in AST and ALT on admission normalized by day three, suggesting minor liver contusion or stress. There were no signs of pancreatic endocrine dysfunction; blood glucose remained normal (70–90 mg/dL). Coagulation and renal function stayed normal. These laboratory findings corroborated an acute traumatic pancreatitis that gradually improved under conservative management.

### 3.3. Imaging Findings

**Figure 3:**
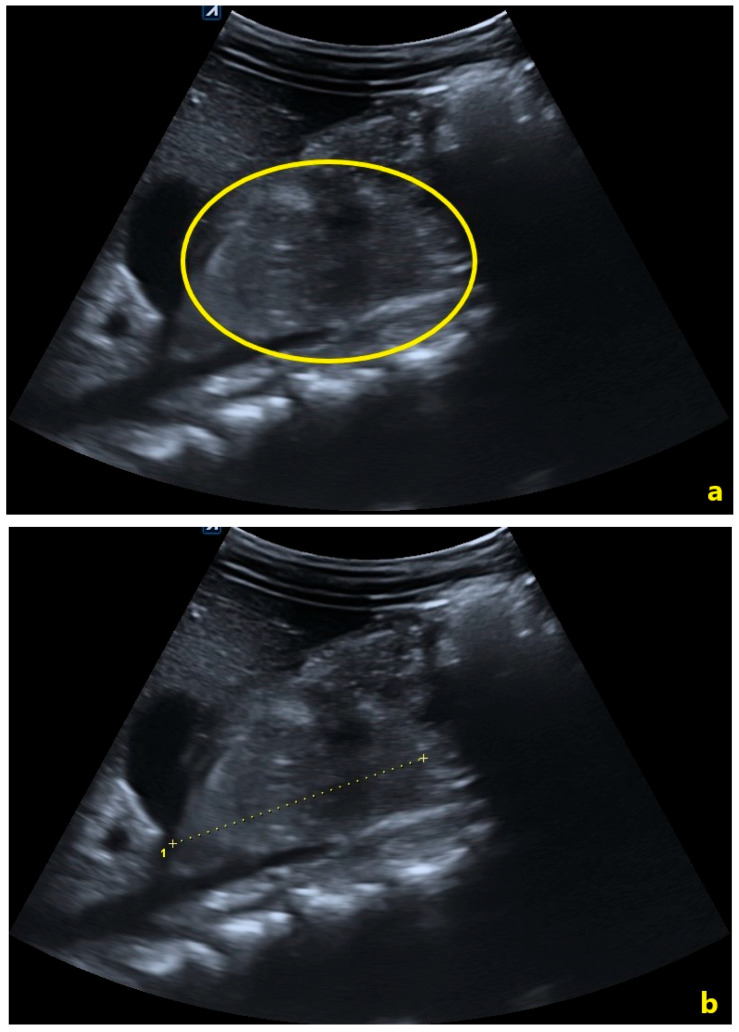
Abdominal ultrasound performed four hours post-injury. (**a**) The yellow oval delineates the heterogeneous hyperechoic pancreatic head, reflecting acute post-traumatic parenchymal changes. No organized fluid collection is identified at this stage. (**b**) Calipers demonstrate the craniocaudal dimension of the edematous pancreatic head, measuring approximately 57 mm.

An abdominal US performed four hours post-injury (Figure 3) did not reveal any solid-organ laceration. However, it identified a subtly heterogeneous, hyperechoic area (42 × 23 × 57 mm TS × AP × CC) in the region of the pancreatic head, with a thin rim of free fluid in the subhepatic space and right iliac fossa raising suspicion of pancreatic contusion.

**Figure 4:**
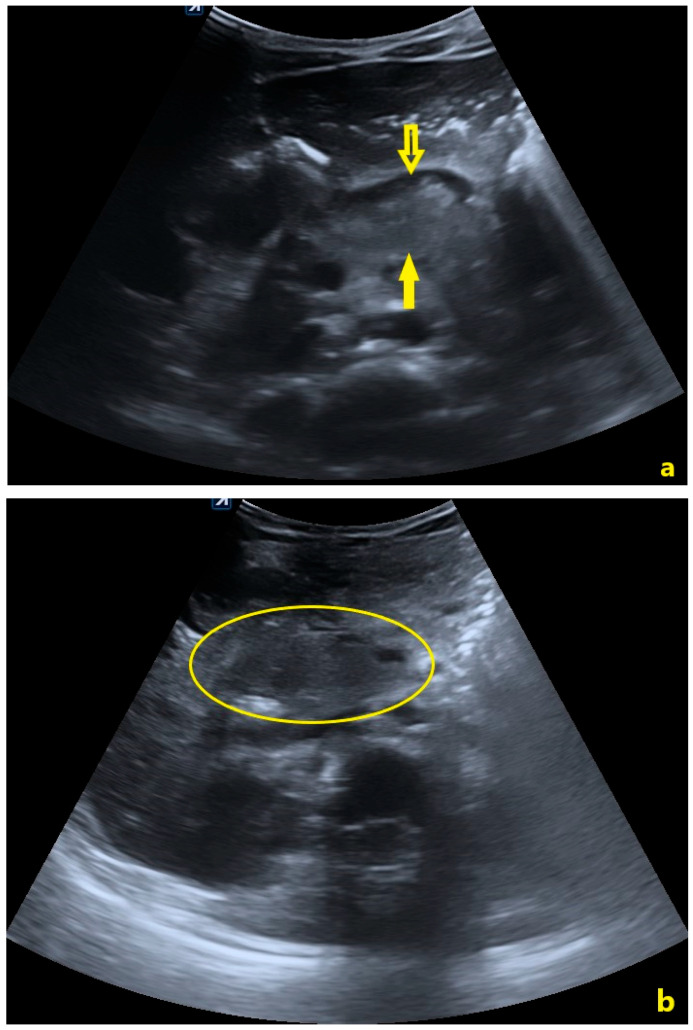

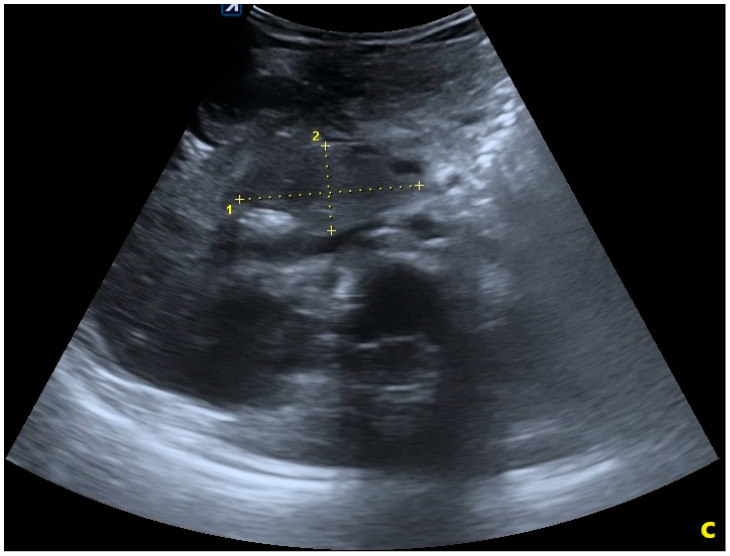
Abdominal ultrasound performed on day 2 post-injury. (**a**) The solid yellow arrow indicates the pancreas. The open yellow arrow identifies peripancreatic fluid in the lesser omental bursa. (**b**) The yellow oval delineates the progressively hypoechoic pancreatic head, reflecting evolving post-traumatic parenchymal changes. (**c**) Calipers delineate the inhomogeneous area within the pancreatic head, measuring 48 × 23 mm (CC × AP).

A repeat US on day two showed persistence of the heterogeneous lesion in the pancreatic head/uncinate region (measuring 48 × 23 mm TS × AP) and small, stable amounts of anechoic fluid in the lesser sac, subhepatic area, pelvis, and right paracolic gutter (all fluid pockets ≤ 1 cm thickness) (Figure 4).

**Figure 5:**
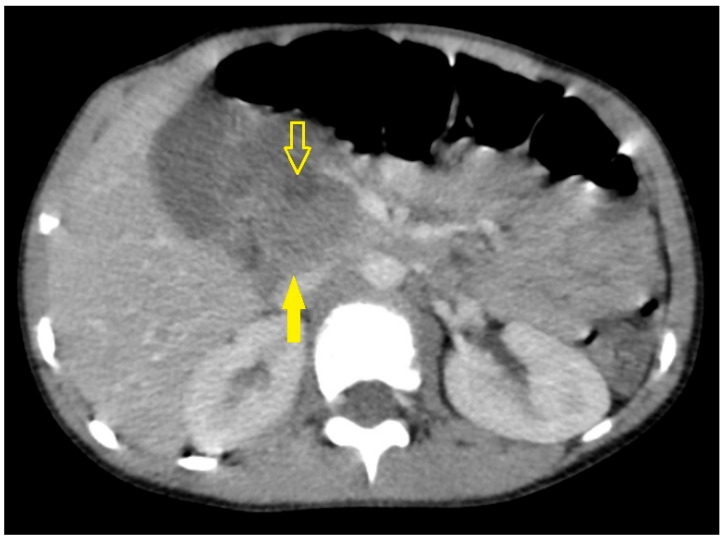
CECT of the abdomen performed on day 4 post-injury. The solid yellow arrow indicates the inhomogeneous uncinate process of the pancreas, reflecting post-traumatic parenchymal changes. The open yellow arrow points to a hypodense non-enhancing peripancreatic fluid collection in the region of the pancreatic uncinate process, consistent with an evolving APFC.

On hospital day four, an abdominal CECT was performed to better characterize the injury. It revealed an enlarged, contused proximal pancreas (head/uncinate) with an internal hypodense area (~20 × 14 mm TS × AP) and poor contrast enhancement, consistent with focal pancreatic injury/contusion (Figure 5).

An adjacent peripancreatic fluid collection (~16 mm at its largest dimension) was noted anterior to the pancreatic head. The distal pancreas (body and tail) appeared normal, and no injuries to other abdominal organs (liver, spleen, kidneys, gastrointestinal tract) were identified. The radiologic impression was a contusion of the pancreatic head/uncinate process with a contained peripancreatic fluid collection, raising the possibility of a developing PPC.

**Figure 6:**
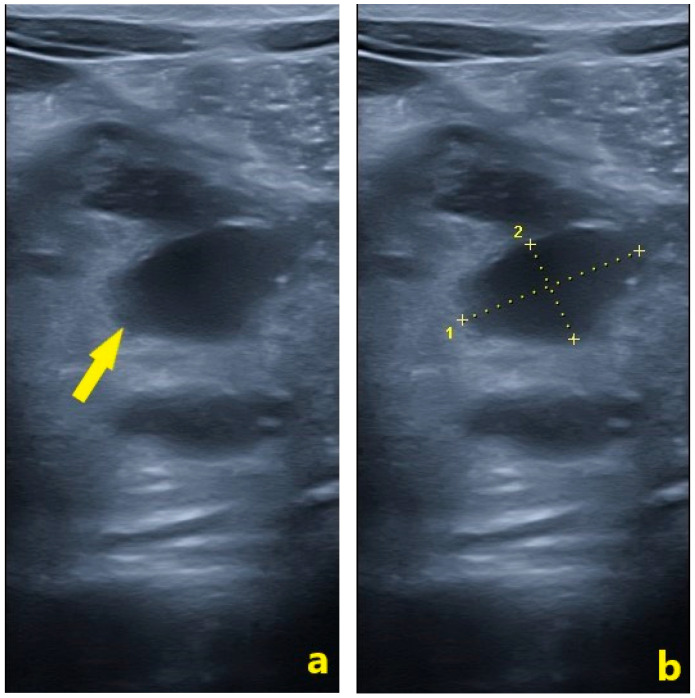
Abdominal ultrasound performed on day 6 post-injury. (**a**) The solid yellow arrow indicates a well-circumscribed anechoic peripancreatic fluid collection anterior to the pancreatic head, consistent with an evolving APFC. (**b**) Calipers indicate the collection’s dimensions: 21 × 12 mm (TS × AP).

Serial US examinations were performed to track the pancreatic injury. By day 6, the uncinate process remained heterogeneous and the known fluid collection persisted, measuring approximately 21 × 12 mm (TS × AP), with no significant new fluid in the abdomen (Figure 6).

**Figure 7:**
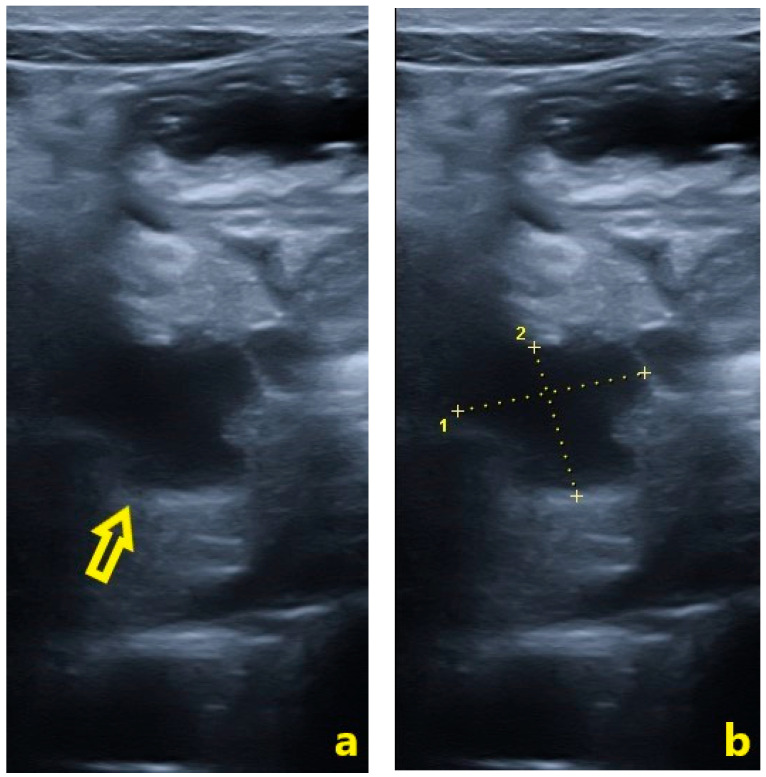
Abdominal ultrasound performed on day 10 post-injury. (**a**) The open yellow arrow indicates a well-defined, homogeneous peripancreatic fluid collection anterior to the pancreatic head, with progressively clearer margins compared to prior examinations, consistent with advancing organization. (**b**) Calipers indicate the collection’s dimensions: 20 × 16 mm (TS × AP).

On day 10, the US showed a well-defined, homogeneous peripancreatic fluid collection anterior to the pancreatic head, measuring ~20 × 16 mm (TS × AP), with minimal pelvic free fluid (~12 mm in the rectovesical recess) (Figure 7).

By day 12, the pancreatic fluid collection had slightly enlarged to ~23 × 22 mm (TS × AP), demonstrating a progressively rounder configuration compared to the irregular morphology observed on day 10, consistent with advancing encapsulation and organizational changes characteristic of evolving pseudocyst formation. It remained well-circumscribed, without signs of abscess or hemorrhage. Small, reactive free fluid pockets were present in dependent areas (pelvis and flanks), likely attributable to local inflammation or minor pancreatic leak.

### 3.4. Hospital Course and Management

The patient was admitted to the pediatric surgery service for observation and NOM. He was kept on bed rest and monitored closely with serial abdominal examinations, ultrasound imaging, and vital sign monitoring to detect any sign of peritonitis or hemodynamic instability. To minimize pancreatic stimulation, the child was kept nil per os (NPO) during the acute phase. He received maintenance IV fluids (crystalloids with dextrose at 100 mL/kg/day) to ensure hydration and basal caloric needs. Partial parenteral nutrition was provided while enteral feeding was withheld. As pain and inflammation subsided over the first week, oral intake was gradually reintroduced, starting with clear liquids and progressing to a low-fat diet. To aid digestion and further reduce pancreatic exocrine activity, pancreatic enzyme supplementation (pancrelipase 500–1000 U/kg per meal) was given with meals once oral feeding resumed.

Adequate analgesia was maintained using non-opioid medications. He received regular paracetamol (acetaminophen 15 mg/kg every 6 h) and metamizole (10 mg/kg every 8 h) for pain and fever control. Drotaverine (2 mg/kg/day in divided doses) was used to relieve abdominal cramps. Opioids were avoided (to prevent sphincter of Oddi spasm and ileus). Ondansetron (0.15 mg/kg per dose) was administered for nausea as needed; vomiting ceased after the initial period. Bowel function was supported with simethicone drops (20 mg per dose, 2–3 times daily) and lactulose syrup (1 mL/kg/day) to prevent constipation.

Subcutaneous octreotide (2 μg/kg every 8 h) was administered to suppress pancreatic exocrine secretion, to promote APFC resolution, and to limit further fluid leakage. Octreotide was dosed according to body weight and continued throughout the hospitalization. An oral proton-pump inhibitor (omeprazole, 20 mg daily) was initiated to protect the gastric mucosa during NPO status and stress, and to potentially reduce duodenal secretin-induced pancreatic stimulation. Broad-spectrum antibiotics were given prophylactically to reduce the risk of infection of the injured pancreas or fluid collection. The regimen included a beta-lactam/beta-lactamase inhibitor (ampicillin–sulbactam, 100 mg/kg/day IV in divided doses, transitioning to oral amoxicillin–clavulanate, 45 mg/kg/day) to cover the gut flora. This was initiated early due to pancreatic injury and a fluid collection, in accordance with institutional practice for PT.

Under this comprehensive conservative care, the child’s condition steadily improved. He never developed signs of peritonitis or sepsis. By hospital day 10, his abdominal pain had markedly decreased, and he was tolerating a light diet with pancreatic enzyme supplementation. Follow-up US demonstrated that the APFC remained small (approximately 2–2.3 cm) and showed no enlargement or complex features. There were no fevers or laboratory indications of abscess formation (WBC and CRP normalized). Thus, no invasive interventions (such as percutaneous drainage or surgical exploration) were necessary. The plan was to allow the APFC to potentially resolve spontaneously, as is often observed in pediatric cases. On day 16, the patient was discharged home in improved condition. At discharge, he was pain-free, tolerating a regular diet, and his pancreatic enzyme levels had declined substantially (though they remained above normal). He was sent home with pancreatic enzyme supplements to take with meals, with octreotide gradually tapered off before discharge, and oral omeprazole for a few more weeks. The family was instructed to maintain a low-fat diet for the child for approximately one month to minimize pancreatic stimulation. They were educated on signs of potential complications (recurrent abdominal pain, vomiting, fever) and advised to seek immediate care if those occurred.

### 3.5. Follow-Up Plans

Arrangements were made for close outpatient follow-up. A repeat abdominal US was scheduled a few weeks post-discharge to monitor the status of the APFC. The pediatric surgery and pediatric gastroenterology teams would jointly follow the patient to ensure resolution of the APFC and to manage any late sequelae (e.g., if APFC enlargement or persistence required intervention). Although clear instructions for follow-up appointments were provided to the parents, the child did not return within 3 months of hospital discharge. This case highlights a favorable short-term outcome with non-operative management of a significant pancreatic injury in a young child.

## 4. Discussion

BAT in children warrants high vigilance even when initial signs are mild [1,2]. The conspicuous upper abdominal bruise (analogous to a “seatbelt sign”) in this case is a red flag indicating significant force; pediatric studies have found that roughly one-third of children with an abdominal wall bruise suffer intra-abdominal injuries on imaging [4,7,8]. Notably, the present case involved an isolated pancreatic injury without associated solid organ or hollow viscus trauma, a relatively uncommon presentation in pediatric BAT.

The laboratory trends in this case are characteristic of acute traumatic pancreatitis that resolved with conservative management. Serum lipase peaked at 6579 U/L on day 12, with intermittent fluctuations throughout, remaining elevated longer than amylase (peak 709 U/L on day 12), consistent with known enzyme kinetics in which lipase typically peaks later and persists longer [2,9,10]. The delayed peak may indicate a transient ductal leak or evolving APFC, which often forms in children weeks after injury and frequently resolves without intervention [11]. Inflammatory markers further supported a mild, self-limiting course: CRP peaked at only 0.8 mg/dL, well below thresholds associated with severe pancreatitis; WBC and liver enzymes normalized quickly; and no evidence of endocrine, renal, or hematologic dysfunction emerged [12,13].

This pattern aligns with evidence supporting NOM in stable pediatric pancreatic trauma cases, even for high-grade injuries [14,15]. Though APFC formation is more frequent with conservative therapy, most are uncomplicated and resolve spontaneously [11]. This child’s enzyme trends, mild inflammation, and uncomplicated recovery reflect the expected trajectory of successful NOM.

In this case, the initial abdominal US revealed a heterogeneous hyperechoic lesion at the pancreatic head with minor peritoneal fluid—findings suspicious for pancreatic contusion. The US has limited sensitivity for detecting pancreatic parenchymal or ductal injuries, particularly when bowel gas obscures retroperitoneal structures, with studies reporting sensitivities of 27–45% in pediatric BAT [11,16,17]. Nevertheless, its safety, portability, and lack of radiation make it a valuable tool for serial monitoring in children.

The CT scan performed on day 4 showed hallmark features of a grade IB pancreatic contusion, showing focal hypodensity and poor enhancement of the head/uncinate process accompanied by a localized peripancreatic fluid collection (20 × 14 mm), which is consistent with well-established imaging signs of contusion [18]. However, CT has limited sensitivity for detecting disruptions of the main pancreatic duct (MPD), with recent pediatric studies reporting detection rates of only 52–54% even with high-resolution scanners [11,16]. Thus, while CT is valuable for identifying parenchymal damage and peripancreatic fluid collections, it cannot reliably exclude ductal trauma [19,20]. Although MRI and MRCP represent the gold standard for MPD evaluation—providing non-invasive, radiation-free ductal assessment that directly informs the decision between conservative and surgical management—they were unavailable at our institution. Given the persistently elevated pancreatic enzyme levels and the need to exclude associated abdominal injuries, CECT was performed as the most reliable available modality. The decision was carefully weighed against the inherent radiation exposure in this 5-year-old patient. It was ultimately justified by the clinical need to exclude ductal disruption and higher-grade injuries, which would have significantly altered management.

In accordance with the revised Atlanta Classification, peripancreatic fluid collections occurring within the first four weeks following acute pancreatitis are designated as APFC, while the term pseudocyst is strictly reserved for collections persisting beyond four weeks with a well-defined inflammatory wall [5]. Serial ultrasound examinations demonstrated progressive evolution from an ill-defined peripancreatic collection at 4 h post-injury to an increasingly well-delineated fluid collection by day 10, consistent with advancing organization and evolving pseudocyst formation. Although follow-up was unavailable, the morphological trajectory observed was consistent with the anticipated evolution toward a mature pancreatic pseudocyst. On CT, pseudocysts typically appear as round or oval, homogeneous low-attenuation collections with smooth borders adjacent to the pancreas [21]. True pseudocyst development usually requires 4–6 weeks as fibrous encapsulation occurs; therefore, the fluid collection noted by day 12 is more accurately considered an evolving APFC [22]. Nonetheless, its well-circumscribed nature and containment without infection were reassuring features supporting NOM.

Serial imaging demonstrated progressive morphological evolution of the peripancreatic fluid collection, with increasing circumscription and encapsulation by day 12, consistent with evolving pseudocyst formation. The absence of complex features, such as abscesses, hemorrhage, or vascular involvement, supported conservative observation [15]. The patient was hemodynamically stable with a grade IB pancreatic injury according to the 2024 revised AAST scale, with no evidence of ductal disruption or infection, making him an appropriate candidate for NOM [23]. Hemodynamic stability and absence of peritoneal signs have repeatedly been associated with favorable outcomes under conservative care in pediatric trauma series [24,25,26]. Kopljar et al. reported NOM success rates of approximately 87% in children with blunt pancreatic trauma, Lyttle et al. observed rates up to 89% even among select higher-grade injuries, and Kaufman et al. emphasized NOM as the preferred initial strategy when ductal injury and hemodynamic compromise are absent [24,25,26].

Imaging criteria also played a key role: CECT showed a parenchymal contusion without MPD disruption, and serial US demonstrated a small, well-circumscribed collection without septations or internal debris. The absence of ductal disruption on high-resolution imaging combined with a stable clinical picture is a validated indication for NOM in children [11,16].

The pancreatic enzyme profile demonstrated a characteristic pattern of post-traumatic pancreatitis. Although a sustained downward trend was not observed within the available follow-up window, the absence of clinical deterioration, peritonitis, or systemic inflammatory response syndrome, combined with the favorable imaging trajectory, supported the interpretation of a self-limiting process. It is well recognized that biochemical normalization of pancreatic enzymes may lag significantly behind clinical and radiological improvement, and enzyme levels alone should not be used as the sole determinant of disease progression or resolution in pediatric pancreatic trauma [19,22].

Recent pediatric studies affirm that stable, asymptomatic pancreatic fluid collections in children can be managed expectantly [11,22]. These findings support the contemporary practice of observing small, stable, afebrile collections without duct disruption or infection with serial imaging rather than intervening early. They are reinforced by evidence showing no improvement in clinical outcomes with routine drainage, a high likelihood of spontaneous resolution, and comparable efficacy of conservative versus interventional management with lower complication rates [19,22].

Early in management, the child was kept NPO with IV fluids to rest the pancreas [27]. In alignment with current guidelines for mild acute pancreatitis, which advocate early oral refeeding rather than prolonged fasting, oral intake was progressively reintroduced once pain and nausea subsided—beginning with clear liquids and advancing to a low-fat diet [28,29,30,31]. Early enteral feeding has been shown to reduce infectious complications and shorten hospital stay in pediatric pancreatitis, and was applied analogously in this post-traumatic setting. PERT was administered to support digestion once oral feeding resumed, though robust evidence for its use specifically in post-traumatic pediatric pancreatitis remains limited [28,32]. Partial parenteral nutrition was provided until oral intake was sufficient, reflecting a shift away from routine TPN toward earlier feeding [22,29]. Symptom control was achieved with a non-opioid multimodal analgesic regimen including paracetamol, metamizole, and drotaverine, consistent with supportive care guidelines [28,33]. Opioids were avoided due to concerns about sphincter of Oddi spasm and ileus [33].

Octreotide was administered to suppress pancreatic exocrine secretion and promote APFC resolution, based on institutional practice and pediatric case series reporting clinical improvement with somatostatin analog therapy, while recognizing that robust evidence from controlled trials is lacking [11,28,34]. A proton-pump inhibitor was given for gastric protection and to reduce pancreatic stimulation, though supporting evidence remains limited [27,35]. Broad-spectrum antibiotics (IV ampicillin–sulbactam, transitioning to oral amoxicillin–clavulanate) were administered empirically in accordance with institutional protocol, acknowledging that evidence for prophylactic antibiotic use in this setting remains inconclusive [27]. No infections developed, supporting the safety of this approach.

Under this conservative regimen, the child improved steadily. He never developed peritonitis or sepsis, a major challenge in intensive care [36]. At discharge on day 16, he was asymptomatic on a regular low-fat diet with enzyme supplements. Also, his labs and clinical status had normalized, although no subsequent follow-up data were available to confirm sustained resolution. In line with current pediatric trauma guidelines, spontaneous pseudocyst resolution would be anticipated with continued conservative management, though radiological confirmation was unavailable in this case [37,38].

This case report has several limitations. As a single case, no causal inferences can be drawn regarding the efficacy of specific interventions; the favorable outcome may reflect the natural history of a low-grade injury rather than any individual treatment component. The use of prophylactic antibiotics and octreotide was guided by institutional protocol rather than robust trial evidence. Additionally, the absence of MRI/MRCP precluded definitive ductal assessment, and the lack of long-term follow-up imaging prevented confirmation of complete pseudocyst resolution.

From a clinical perspective, this case underscores several important lessons in pediatric blunt abdominal trauma. Epigastric bruising should prompt a high index of suspicion for pancreatic injury, even in hemodynamically stable children with initially subtle imaging findings. Serial monitoring of pancreatic enzymes and repeated ultrasound examinations are valuable in detecting evolving pancreatic collections that may not be evident on early imaging. Importantly, small, asymptomatic post-traumatic pancreatic pseudocysts in stable patients can be safely managed conservatively with close observation, avoiding unnecessary invasive interventions. Together, these principles support a structured, cautious approach to pediatric pancreatic trauma that prioritizes careful monitoring over routine operative management.

## 5. Conclusions

This case demonstrates the successful non-operative management of isolated post-traumatic pancreatitis with an evolving peripancreatic fluid collection in a hemodynamically stable 5-year-old following blunt abdominal trauma. Serial imaging documented the dynamic evolution of the collection, and the patient improved steadily without surgical or percutaneous intervention. In accordance with the 2024 revised AAST classification, this grade IB injury was managed conservatively with bowel rest, supportive care, octreotide, and close monitoring—consistent with current pediatric literature supporting NOM for hemodynamically stable patients with pancreatic injuries without ductal disruption. This case reinforces that carefully selected pediatric pancreatic injuries can be managed safely without surgery, with structured follow-up and avoidance of unnecessary invasive procedures, achieving excellent outcomes while minimizing morbidity.

## Figures and Tables

**Table 1:** Laboratory findings.

Parameter	Day 1	Day 3	Day 6	Day 9	Day 12	Day 16
WBC (×10^3^/µL)	14	7.83	7.02	6.9	5.9	5.1
Hemoglobin (g/dL)	12.4	13.0	12.39	12.6	12.6	12.6
Hematocrit (%)	34.9	38.7	39.2	36	36	36
Platelets (×10^3^/µL)	347	307	359	455	365	380
Neutrophils (×10^3^/µL)	12.3	4.3	3.3	3.25	3.0	2.8
Lymphocytes (×10^3^/µL)	1.1	2.7	2.6	2.6	2.2	2.1
Urea (mg/dL)	29	21	22	32	28	20
Creatinine (mg/dL)	0.3	0.3	0.27	0.3	0.37	0.32
Glucose (mg/dL)	80	78	70	90	82	86
ALT (U/L)	48	29	22	26	23.5	21
AST (U/L)	67	37	32	38	37	40
Total Bilirubin (mg/dL)	0.5	0.76	0.73	0.55	0.74	0.84
Alkaline Phosphatase (U/L)	213	212	240	227	232	180
CK (U/L)	139	65	74	82	80.5	78
LDH (U/L)	286	261	160	175	234	246
APTT (s)	19.8	22.9	23.0	23.2	22.8	22.7
INR	0.98	1.0	0.95	0.98	0.96	1.0
Fibrinogen (mg/dL)	178	209	212	221	250	196

Reference range for laboratory tests: White blood cell count (WBC) 4–12 × 10^3^/µL; Hemoglobin 11.5–14.5 g/dL; Hematocrit 33–43%; Platelets 150–450 × 10^3^/µL; Neutrophils 1.5–8 × 10^3^/µL; Lymphocytes 3–6.5 × 10^3^/µL; Urea 11–45 mg/dL; Creatinine 0.2–0.5 mg/dL; Glucose 60–110 mg/dL; Alanine aminotransferase (ALT) 10–25 U/L; Aspartate transferase (AST) 21–44 U/L; Total Bilirubin 0.3–1.2 mg/dL; Alkaline Phosphatase 150–380 U/L; Creatine Kinase (CK) 74–230 U/L; Lactate dehydrogenase (LDH) 155–345 U/L; Activated partial thromboplastin time (APTT) 22–37 s; International Normalized Ratio (INR) 0.94–1.13; Fibrinogen 150–400 mg/dL.

## Data Availability

The original contributions presented in this study are included in the article. Further inquiries can be directed to the corresponding author.

## Abbreviations

The following abbreviations are used in this manuscript:

PT	Pancreatic trauma
PPC	Pancreatic pseudocyst
US	Ultrasound
CECT	Contrast-enhanced computed tomography
PERT	Pancreatic enzyme replacement therapy
APFC	Acute peripancreatic fluid collection
BPI	Blunt pancreatic injury
NOM	Non-operative management
BAT	Blunt Abdominal Trauma
WBC	White blood cell count
CRP	C-reactive protein
ALT	Alanine aminotransferase
AST	Aspartate transferase
CK	Creatine Kinase
LDH	Lactate dehydrogenase
APTT	Activated partial thromboplastin time
INR	International Normalized Ratio
NPO	Nil per os
MPD	Main pancreatic duct
MRI	Magnetic resonance imaging
MRCP	Magnetic Resonance Cholangiopancreatography
TPN	Total parenteral nutrition
AAST	American Association for the Surgery of Trauma
